# Factors predicting one-year mortality in amyotrophic lateral sclerosis patients – data from a population-based registry

**DOI:** 10.1186/s12883-014-0197-9

**Published:** 2014-10-04

**Authors:** Joachim Wolf, Anton Safer, Johannes C Wöhrle, Frederick Palm, Wilfred A Nix, Matthias Maschke, Armin J Grau

**Affiliations:** Department of Neurology, Klinikum der Stadt Ludwigshafen, Bremserstraße 79, Ludwigshafen, 67063 Germany; Institute of Public Health, Medical Faculty, Ruprecht-Karls-University, Heidelberg, Germany; Department of Neurology, Katholisches Klinikum, Brüderhaus, Koblenz, Germany; Department of Neurology, Universitätsmedizin, Mainz, Germany; Department of Neurology, Krankenhaus der Barmherzigen Brüder, Trier, Germany

**Keywords:** Amyotrophic lateral sclerosis, Mortality rate, Prognostic factors, Population register

## Abstract

**Background:**

Survival in amyotrophic lateral sclerosis varies considerably. About one third of the patients die within 12 months after first diagnosis. The early recognition of fast progression is essential for patients and neurologists to weigh up invasive therapeutic interventions. In a prospective, population-based cohort of ALS patients in Rhineland-Palatinate, Germany, we identified significant prognostic factors at time of diagnosis that allow prediction of early death within first 12 months.

**Methods:**

Incident cases, diagnosed between October 2009 and September 2012 were enrolled and followed up at regular intervals of 3 to 6 months. Univariate analysis utilized the Log-Rank Test to identify association between candidate demographic and disease variables and one-year mortality. In a second step we investigated a multiple logistic regression model for the optimal prediction of one-year mortality rate.

**Results:**

In the cohort of 176 ALS patients (mean age 66.2 years; follow-up 100%) one-year mortality rate from diagnosis was 34.1%. Multivariate analysis revealed that age over 75 years, interval between symptom onset and diagnosis below 7 months, decline of body weight before diagnosis exceeding 2 BMI units and Functional Rating Score below 31 points were independent factors predicting early death.

**Conclusions:**

Probability of early death within 12 months from diagnosis is predicted by advanced age, short interval between symptom onset and first diagnosis, rapid decline of body weight before diagnosis and advanced functional impairment.

**Trial registration:**

ClinicalTrials.gov (NCT01955369, registered September 28, 2013)

## Background

Amyotrophic lateral sclerosis (ALS) is a severe neurodegenerative disease with a progressive decline of upper and lower motor neurons leading to disability and death. Survival in ALS is highly variable, with a wide range from a few months to many years. Population-based prospective registries report one-year mortality rates after diagnosis ranging from 22% [1,2] to 34% [3]. Early identification of a potentially malignant variant of ALS would be essential for patients to take vital decisions, and might help patients in cooperation with their neurologists to decide in favour or against possibly burdensome invasive therapeutic interventions, like percutaneous endoscopic gastrostomy or mechanical ventilation.

Previous population-based ALS registries identified different prognostic factors of overall survival including demographic factors (age at onset, gender), clinical factors (e.g. type of onset, rate of disease progression), and factors related to nutrition or respiration [4].

Our study is based on a prospective, population-based cohort of ALS patients. The aim of our paper is to identify prognostic factors at time of diagnosis related to fast disease progression and early death within 12 months after first diagnosis.

## Methods

In 2009 a prospective and population-based ALS registry was established in Rhineland-Palatinate, a state in the south-west of Germany with 4 million inhabitants (census data 2011: n = 3989800, 1950420 men, and 2039380 women) [5]. Newly diagnosed ALS patients in the period October 2009 to September 2012 were enrolled and followed-up at regular intervals of 3 to 6 months. Eligible patients had a minimum age of 18 years. We used multiple overlapping sources of information to ensure completeness of case ascertainment. The establishment of the ALS registry Rhineland-Palatinate has recently been described [6].

The registry has been approved by the data protection commissioner and by the local ethics committee of Rhineland-Palatinate (registration-number 837.253.09). It has been registered in the publicly accessible trial register ClinicalTrials.gov (NCT01955369). According to legal requirements, patients could be registered at the data holding centre (Department of Neurology, Klinikum Ludwigshafen), including personal data, if written informed consent signed by patient or the legal representative was available. In the absence of written informed consent, a basic data set (demographic and phenotypic data) was generated with the use of a pseudonymisation code, a procedure to avoid double registration.

We used the revised El Escorial criteria (EEC) [7] to classify cases into four categories of disease likelihood: possible ALS, probable ALS with laboratory confirmation, probable respectively definite ALS, depending on the presence of upper and lower motor neuron involvement in defined body regions. Patients with a pure lower or a pure upper motor neuron disease were excluded. Functional impairment was assessed by the ALS Functional Rating Scale (FRS) in the initially published version [8]. Minimum follow-up period for this paper was 18 months. All times to death have been observed, and thus there was no censoring of observations. To determine the causes of death we used information of attending physicians, neurologists and family members as well as data of death certificates from local health authorities.

### Statistical analysis

One-year mortality rate was tested for independence univariately with all candidate factors for survival prediction using the Log-Rank Test (LRT). Continuous variables were subdivided into categories by one or more cut-points with commonly accepted values or, if not applicable, with groups of approximately equal numbers. This procedure was applied to age (<65, 66–75, >75 years), gender (male, female), site of onset (bulbar, spinal), site of spinal onset (upper limbs, lower limbs, upper and lower limbs, trunk), delay between symptom onset and diagnosis (DOD, 0–6, 7–12, 13–24 months, ≥25 months), body-mass-index (BMI) (<25, ≥25 kg/m2), difference between BMI at diagnosis and six months before according to patients’ statement (Diff-BMI, <1, 1- < 2, ≥2), EEC (possible, probable with laboratory confirmation, probable, definite), FRS (>32, ≤32, and subdivided into quintiles: <27, 27–30, 31–33, 34–36, 37–40), forced vital capacity (FVC, <80, ≥80%), creatine kinase (CK, <150, ≥150 U/l), pCO2 (<42, ≥42 mmHg), previous alcohol consumption status (no, yes), previous smoking status (no, yes), and social life (single person, living in a partnership). Time was measured in months from date of diagnosis to death or to tracheostomy, and categorized to a binary survival variable (1 = death within 12 months; 0 = survival > 12 months).

We predicted 1-year death rate by a multiple logistic regression model from those demographic and disease variables at time of diagnosis that were identified as statistically significant. Starting with the maximum number of predictors, a stepwise selection procedure was used to eliminate the non-significant variables from the full model. Model fit was described by LRT for the global Null hypothesis (all regression coefficients β =0). R^2^ is the coefficient of determination describing the percentage of total variability being explained by the model.

The relationship between mortality rate one year after diagnosis and FRS at diagnosis is visualized by a bubble plot, with the estimated logistic regression function predicting% mortality rate from FRS. The area of the bubbles is proportional to the number of observations.

P-values ≤0.05 were considered significant. All data were analysed using SAS 9.3 software (SAS Institute, North Carolina), PROC LIFETEST being used for the Log-Rank Test, and PROC LOGISTIC for the logistic model predicting 12-month mortality rate.

## Results

During the three year surveillance period 200 incident ALS patients (106 males, 94 females) were enrolled. 24 patients (10 males, 14 females) were lost during early follow-up (17 patients after date of diagnosis, 7 patients after first follow-up), and therefore excluded. Demographic data of the excluded patients didn’t differ from those 176 patients, which have been tracked for status 12 months after first diagnosis.

Mean age at diagnosis was 66.2 years (SD 10.3, range 23–85, median 68), in men 65.1 years (range 23–85, median 67), and in women 67.4 years (range 30–85, median 70). Mean DOD was 12.5 months (SD 12.8, median 9). One year mortality rate from diagnosis was 34.1% (N = 60). The causes of death regarding patients who had died within 12 months from first diagnosis encompassed respiratory failure due to weakness of breathing muscle pump (n = 34; 56.7%) and due to pneumonia (n = 7; 11.7%), death from cachexia and marasmus (n = 6; 10%), and cardiovascular causes of death including sudden death (n = 4; 6.7%). In 9 patients the causes of death could not be determined (15%).

### Survival analysis

Table 1 shows the results of categorical analysis of tested variables potentially related to rapid decline and early mortality in ALS. Due to complete tracking over the first year after ALS diagnosis all survival times are observed, and none is censored.

**Table 1:** **Univariate analysis of potential prognostic factors associated with mortality one year after first diagnosis** (**N** = **176**)

**Parameters at FD**	**N**		**Category**	**Total**	**Death during 12 months from FD**		**Log-** **rank test**	
	**ob**-**served**	**mis**-**sing**			**n = ** **60**	**%**	**X** ^**2**^ **(DF)**	**p** **(X** ^**2**^ **)**
**Age**	176	0	≤ 65	69	18	26%	24.13 (2)	< **0.0001**
			66-75	84	25	30%		
			> 75	23	17	74%		
**Gender**	176	0	male	96	31	32%	0.29 (1)	0.59
			female	80	29	36%		
**Site of onset**	176	0	bulbar	62	25	40%	1.71 (1)	0.19
			spinal	114	35	31%		
**Site of spinal onset**	114	0	upper limbs	39	10	26%	2.58 (4)	0.64
			lower limbs	60	20	33%		
			upper + lower limbs	8	3	38%		
			trunk	7	2	29%		
**DOD** **[months]**	176	0	0 - 6	60	27	45%	9.41 (3)	**0.024**
			07-12	61	17	28%		
			13-24	37	15	41%		
			≥ 25	18	1	6%		
**BMI** **[kg/** **m** ^**2**^ **]**	173	3	< 25	92	34	37%	0.97 (1)	0.33
			≥ 25	81	24	30%		
**Diff**-**BMI** **[kg/** **m** ^**2**^ **]**	173	3	< 1	87	14	16%	33.14 (2)	< **0.0001**
			1 - < 2	31	11	35%		
			≥ 2	55	33	60%		
**EEC**	176	0	possible	31	6	19%	16.21 (3)	**0.001**
			probable (lab. conf.)	54	12	22%		
			probable	63	26	41%		
			definite	28	16	57%		
**FRS**	173	3	< 32	72	44	61%	46.92 (1)	< **0.0001**
			≥ 32	101	14	14%		
**FVC [%]**	153	23	< 80	31	19	61%	28.61 (1)	< **0.0001**
			≥ 80	122	27	22%		
**PCO**_**2**_ **[mm Hg]**	134	42	< 42	112	33	29%	15.97 (1)	< **0.0001**
			≥ 42	22	14	64%		
**CK** ** [U/** **l]**	153	23	< 150	73	27	37%	1.29 (1)	0.26
			≥ 150	80	24	30%		
**Pre**-**alcohol cons. status**	149	27	no	62	26	42%	2.99 (1)	0.084
			yes	87	19	22%		
**Pre**-**smoking status**	151	25	no	85	23	27%	0.69 (1)	0.41
			yes	66	23	35%		
**social life**	153	23	living in a partnership	109	28	26%	1.59 (1)	0.21
			single person	44	19	43%		
**Abbreviations**	DOD	delay between onset and diagnosis			FD	first diagnosis		
	BMI	body mass index			FRS	functional rating scale		
	Diff-BMI	difference between BMI at diagnosis and six months before			FVC	forced vital capacity		
					PCO_2_	carbondioxide pressure in blood		
	EEC	revised El Escorial criteria			CK	creatinine kinase		

**Figure 1:**
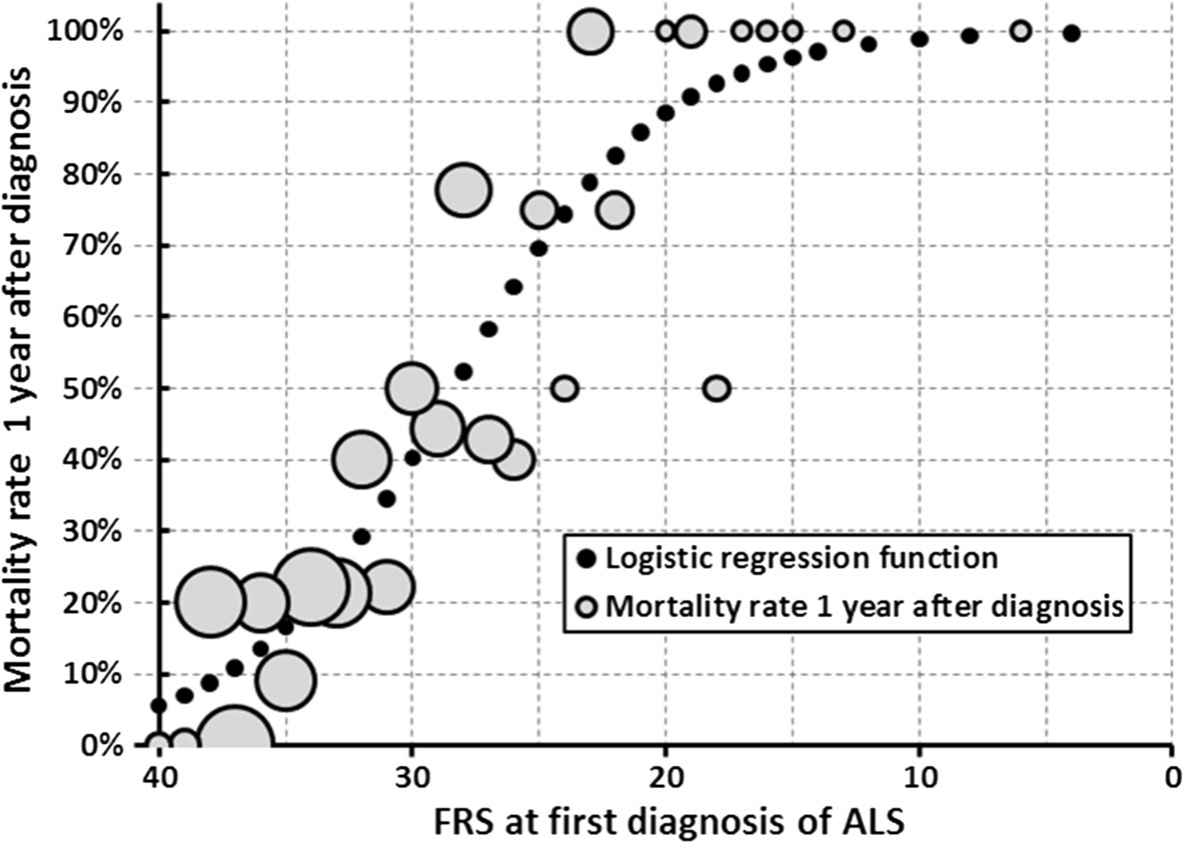
**Mortality rate one year after first diagnosis (FD) of ALS by FRS at FD.** Observed rates: bubble area proportional to number of observations; black dots: logistic function estimate.

In univariate analysis death within 12 months was significantly influenced by several variables. Age over 75 years, short DOD (≤6 months), Diff-BMI ≥ 2, low FRS (<32), low FVC (<80%), pCO_2_ over >42 mmHg and level of diagnostic certainty according to EEC being probable or definite were associated with a prognosis of increased probability of death one year after diagnosis. A slight but not significant trend was observed for previous consumption of alcohol with lower mortality rate (p = 0.08). The strongest association between 12-month mortality and candidate predictors was found with FRS (maximum-scaled coefficient of determination R^2^ = 0.368) (Figure 1).

The final multiple logistic model retained four factors with significant influence on 12-month mortality rate: advanced age at diagnosis (with 3 discrete age categories), and the predictors DOD, Diff-BMI and FRS (Table 2).

**Table 2:** **Multiple logistic regression model predicting the one**-**year mortality rate in ALS patients** (**N** = **176**)

**Parameter**	**Category**	**DF**	**Estimate b**	**Standard error** **(b)**	**Wald X** ^**2**^	**p**	**OR** ** (95% ** **CI)**
**Intercept**		1	−2.323	0.702	10.93	**0.001**	
**AGE**	<=**65**		0.000	Reference			1
	**66**-**75**	1	0.121	0.473	0.07	0.80	1.13 (0.45 - 2.85)
	**76** +	1	1.822	0.709	6.60	**0.01**	**6.2** (1.5 - 25)
**DOD**	**0**-**6**		0.000	Reference			1
	**7**-**12**	1	−0.866	0.523	2.74	0.10	0.42 (0.15 - 1.17)
	**13**-**24**	1	−0.808	0.594	1.85	0.17	0.45 (0.14 - 1.4)
	**25**+	1	−2.999	1.151	6.78	**0.01**	**0.050 **(0.005 - 0.48)
**Diff**_**BMI**	<**1**		0.000	Reference			1
	**1**- < **2**	1	0.227	0.591	0.15	0.70	1.26 (0.39 - 4.0)
	> = **2**	1	1.038	0.512	4.11	**0.04**	**2.8** (1.04 - 7.7)
**FRS**	**Quintile 1: ** **37**-**40**		0.000	Reference			1
	**Quintile 2: ** **34**-**36**	1	0.588	0.796	0.55	0.46	1.8 (0.38 - 8.6)
	**Quintile 3: ** **31**-**33**	1	0.953	0.795	1.47	0.23	2.6 (0.55 - 12)
	**Quintile 4: ** **27**-**30**	1	2.556	0.786	10.57	**0.001**	**12.9** (2.8- 60)
	**Quintile 5: ** **00**-**26**	1	3.520	0.823	18.28	<.**0001**	**33.8** (6.7 - 170)
**Testing the global null hypothesis: ** **BETA= ** **0**							
**Likelihood ratio test**		11			87.67	<.**0001**	
**R** ^**2**^		0.543					

*Abbreviations:* *OR* odds ratio, *CI* confidence interval, *R* ^*2*^ coefficient of determination.

Increased mortality rate was predicted by age: the odds ratio (OR) of one-year mortality rate was 6.2 (95% CI: 1.5 – 25; p = 0.01) for patients over 75 years compared to those diagnosed at ≤65 years. Increased mortality rate was also linked to short DOD. There was a non-significantly reduced OR of 0.42 for DOD 7 – 12 months resp. 0.45 for DOD 13 – 24 months, compared to DOD up to 6 months. Such patients with DOD over 24 months showed a significantly reduced OR of 0.05 (95% CI: 0.005 – 0.48; p = 0.009). These are the best candidates for longer term survival. Loss of at least 2 BMI units during the 6 months preceding diagnosis was linked to a significant increase of OR (2.8, 95% CI: 1.04 – 7.7; p = 0.04), compared to loss of weight less than one BMI unit. Low FRS was linked to high probability of one-year mortality, as visualized by Figure 1. In the multiple logistic model the separation of FRS into quintiles (reference: quintile 1: 37 – 40) showed a strictly increasing OR by quintiles 2 to 5 with significantly increased OR for quintile 4 (FRS 27 – 30; OR 12.9, 95% CI: 2.8 – 60; p = 0.001) and quintile 5 (FRS ≤26; OR 33.8, 95% CI: 6.7 – 170; p < 0.0001).

## Discussion

In contrast to previous studies which analysed prognostic factors with regard to overall survival [2-4,9] we focussed our analysis on variables predicting one-year mortality after first diagnosis in a population-based prospective cohort of 176 ALS patients in Rhineland-Palatinate, Germany.

In this cohort one-year mortality rate was 34%, which is in the upper range of results of previous population-based registries [1-3]. The categorized variables age over 75 years, interval between symptom onset and diagnosis up to 6 months, decline of body weight of 2 or more BMI units during 6 months preceding diagnosis and progressed functional impairment (FRS ≤30) were significant and independent predictors of mortality rate one year after diagnosis. The FRS at time of first diagnosis showed the strongest prognostic value for risk of death, explaining about 37% of the total variance, while the four-factor model explains about 54%.

In concordance with findings of other population-based studies the age at diagnosis was a strong prognostic variable [2,3,10]. Elder patients over 75 years showed distinctly elevated one-year mortality by a factor of about 6 compared to younger patients up to 65 years. In comparison to the local population over 75 years with one-year mortality risk of 7.8% for men and 4.6% for women [5] there was a more than ten-fold elevated one-year mortality risk both for male and female ALS patients over 75 years (81.1% resp. 66.7%).

Several authors postulated that patients with an aggressive disease form sought medical help earlier and diagnostic process was easier and therefore faster [2,3,10-12]. This fact might also explain the shortened latency between onset and diagnosis and the linkage to early death in our study.

Nutritional status deteriorates during the course of disease, and a detrimental nutritional status at diagnosis seems to be associated with increased mortality [13]. In our study crude BMI at diagnosis was no marker of an elevated risk of death, but a marked decrease of BMI within a 6 months interval preceding diagnosis (> = 2 BMI units) predicted a fatal disease progression. Corresponding to a previous study finding [14], we conclude that weight loss at diagnosis but not body weight or BMI was another relevant prognostic factor. However the information of BMI 6 months prior to diagnosis was based on patients’ statements, and therefore susceptible to inaccuracy.

Additionally, we found that a low FRS score at diagnosis indicating an advanced functional impairment was a clear marker of increased probability of death during first year after diagnosis (Figure 1). Similar results both for the ALSFRS [15,16], which we used, and for the revised version (ALSFRS-R) have been previously identified in hospital based studies [17,18].

The El Escorial criteria are a structured tool to define the diagnostic certainty of ALS in individual patients. Although mainly intended for use in research settings [4], several studies could demonstrate that EEC are a valid prognostic marker [2,10], and that the level of definite ALS at diagnosis had a worse prognosis than other EEC levels. In contrast, Zoccolella et al. [9] found no clear correlation between EEC and survival. In our study we used the revised version of the EEC [7]. In multivariate analysis we could not confirm the finding that criteria defining higher levels of certainty (definite, probable ALS) and therefore indicating a more widespread clinical involvement pointed to a worse prognosis within 12 months from diagnosis. It might be possible that only analyses of overall survival with long observation period reveal prognostic effects of EEC. This explanation might also be valid for factors reflecting respiratory status at diagnosis (FVC, pCO_2_) or site of onset. Respiratory variables had no prognostic relevance concerning one-year mortality in our study. However, with regard to the whole course of the disease, FVC < 75% was associated with a poor prognosis in a clinic-based cohort of 1034 patients [19]. The site of onset did not influence risk of early death within 12 months from first diagnosis, whereas other population-based studies found worse prognosis in bulbar onset ALS [3,9,10].

The major weaknesses of our study material are the limited number of patients in the registry, and the resulting lack of statistical power to detect possibly important factors of minor strength.

Despite of the limited number of patients this study has several strengths. The population-based prospective approach is representative of the overall ALS population. Thus data on survival as well as the analysis of prognostic factors are based on all ALS cases and are least likely to be affected by bias [1]. Here we captured a multitude of possibly relevant variables and restricted our point of view to early mortality within 12 months from diagnosis.

## Conclusions

Our results confirm that there are several valid predictors of one-year mortality rate in ALS even at time of diagnosis: age over 75 years, short interval between symptom onset and diagnosis (≤6 months), rapid decline of body weight before diagnosis (≥2 BMI units within 6 months) and advanced functional impairment (FRS ≤ 30 points). These predictor variables of one-year mortality are easy to assess and have the potential to support neurologists and patients in their further therapeutic decisions.
